# Prevalence of non-alcoholic fatty liver disease and its associated factors in individuals with type 1 diabetes: a cross-sectional study in a tertiary care center in Brazil

**DOI:** 10.1186/s13098-021-00649-0

**Published:** 2021-03-19

**Authors:** Bianca Senger Vasconcelos Barros, Fernanda Cruz Monteiro, Carlos Terra, Marilia Brito Gomes

**Affiliations:** 1 Department of Internal Medicine, Diabetes Unit, Pedro Ernesto Hospital, State University of Rio de Janeiro (UERJ), 20.551-030, Boulevard 28 de Setembro, 77 - 3º andar - Vila Isabel, Rio de Janeiro, RJ CEP 20551-030 Brazil; 2 Department of Radiology, Pedro Ernesto Hospital, State University of Rio de Janeiro (UERJ), 20.551-030, Rio de Janeiro, RJ Brazil; 3 Department of Gastroenterology, Pedro Ernesto Hospital, State University of Rio de Janeiro (UERJ), 20.551-030, Rio de Janeiro, RJ Brazil; 4 Department of Gastroenterology, Federal Hospital of Lagoa, Rio de Janeiro, RJ 22470-050 Brazil

**Keywords:** Type 1 diabetes, Fatty liver, NAFLD, Steatosis, Elastography, Ultrasound, Metabolic syndrome

## Abstract

**Background:**

Data on non-alcoholic fatty liver disease (NAFLD) in individuals with type 1 diabetes (T1D) is controversial and so far, there are no published data on the Brazilian population. We investigated the prevalence of steatosis and hepatic fibrosis in a population with T1D from a tertiary care center in Brazil and its associated factors.

**Methods:**

Ninety-five participants with T1D, aged 39 ± 13 years, with disease duration of 21 ± 9 years, being 55 (57.9%) females, from a university hospital in Rio de Janeiro, were screened for NAFLD with hepatic ultrasound (US) and transient elastography (TE).

**Results:**

Prevalence of steatosis was, respectively, 12.6% and 16.8% when US and TE were used for diagnosis of NAFLD. Fibrosis was present in 8.4% of participants. A total of 31.6% of participants had at least one of the hepatic exams altered, which was associated with higher body mass index, waist circumference, hip circumference and waist-to-hip ratio,, presence of metabolic syndrome and higher triglycerides levels, even within the normal range. After multivariate analysis, presence of steatosis was only associated with metabolic syndrome and its component, triglycerides.

**Conclusion:**

In our study, prevalence of NAFLD in ultrasound approximates the one found with TE. Fibrosis was not frequent. Screening should be reserved for participants with T1D and metabolic syndrome, as this was the main factor associated with NAFLD. Triglycerides levels were the only component of metabolic syndrome associated with steatosis. Further studies are necessary to determine the best screening strategy for NAFLD in individuals with T1D. Also, predisposing factors for development in fibrosis in T1D should be further explored in prospective studies.

## Introduction

Non-alcoholic fatty liver disease (NAFLD) is one the most frequent liver diseases and it is associated with obesity, insulin resistance, type 2 diabetes, enhanced cardiovascular risk, and risk for hospitalization and death due to liver complications such as cirrhosis and hepatocellular carcinoma [1]. NAFLD involves a range of alterations including steatosis, steatohepatitis, fibrosis, and cirrhosis [2]. Fibrosis is a marker for the development of hepatic complications and should therefore be assessed to determine the NAFLD prognosis [3]. Global prevalence of NAFLD is around 25% [1, 2, 4]. The presence of steatosis suggests the diagnosis of NAFLD, in the absence of other causes of hepatopathies. Steatosis can be detected by ultrasound, magnetic resonance imaging and, the gold-standard method, liver biopsy [5]. Transient elastography (TE) can also detect steatosis but it is not recommended as first line imaging method and is usually reserved for fibrosis assessment [6, 7]. Although we have a worldwide overweight and obesity epidemic [8] which includes individuals with type 1 diabetes (T1D) [9], NAFLD has not been the focus of many studies with T1D, resulting in a broad range of prevalence from 8 to 53% [2, 10–15]. A recent meta-analysis of Vries et al found a prevalence of 19.3% of NAFLD in T1D and of 22% when only adults with T1D were selected [16]. However, only 20 studies were included in this meta-analysis, resulting in high heterogeneity, attributed to different diagnostic methods used and reinforcing the controversial aspects of this subject in T1D.

Although portal hyperinsulinemia and insulin resistance have been implicated in the development of NAFLD, the pathogenesis in T1D is controversial. In these individuals, exogenous insulin is administered and it achieves high peripherical concentration but low portal concentration. This may prevent hepatic lipogenesis and development of NAFLD in T1D [10, 17]. However, alternative pathogenic pathways, such as activation of lipogenesis in hyperglycemic states and increased flux of fatty acids to the liver due to peripheral insulin resistance and peripheral hyperinsulinemia, may explain how NAFLD could be also a complication in T1D [18].

The aim of this study was to determine the prevalence of steatosis and hepatic fibrosis by two methods, ultrasound and transient elastography, and its associated factors in a population with T1D from a tertiary care center in Brazil and its associated factors.

## Subjects, materials, and methods

### Study design

This was a cross-sectional study conducted between 2016 and 2020, with individuals with T1D, treated by an endocrinologist in the Diabetes Unit at Policlínica Piquet Carneiro, a public tertiary health center. They were consecutively invited to participate in the study during regular visits. We included individuals with T1D, aged at least 13 years old, diagnosed by a physician through classical clinical findings (hyperglycemia, polyuria, weight loss, polydipsia, polyphagia and dependency on insulin therapy since diagnosis), that were assisted for at least 6 months in our center. The exclusion criteria were: being pregnant or breastfeeding at the time of inclusion; known liver disease; daily alcohol ingestion above 20 g for women or 30 g for men; acute infectious process, hospitalization, or ketoacidosis in the 3 months prior to recruitment. All participants or their caregivers signed informed consent and study was approved by local ethics committee.

## Data collection

Data were collected on gender, current age, diabetes duration, years of school attendance, self-reported color-race (White, Black, Brown, Asian or Indigenous, as recommended by Brazilian Institute of Geography and Statistics) [19], alcohol consumption, type of insulin and daily dose, use of other medications and comorbidities. Clinical variables included weight (in kilograms), height (in centimeters), body mass index (BMI), blood pressure (BP), waist circumference (WC; determined at half the distance between the last costal arch and the iliac crest), hip circumference (HC), and random capillary glucose. Overweight was defined by BMI ≥ 25 kg/m^2^ and < 30 kg/m^2^ and obesity was defined by BMI ≥ 30 kg/m^2^. Laboratory measurements were obtained, after an overnight fast: fasting plasma glucose, glycated hemoglobin A1c (HbA1c; measured with high- performance liquid chromatography), urea, creatinine, total cholesterol, high- density lipoprotein cholesterol (HDL), triglycerides, low-density lipoprotein cholesterol (LDL) calculated by Friedewald’s equation, alanine aminotransferase (ALT), aspartate aminotransferase (ALT), ultrasensitive C-reactive protein (CRP), creatine phosphokinase (CPK), gamma-glutamyl transferase (GGT) and uric acid. For ALT and AST, we considered normal values of < 25 U/l for women and < 33 U/l for men [20]. Estimated glomerular filtration rate (eGFR) was calculated with CKD-EPI formula. Fatty liver index (FLI) was calculated with original formula to determine the risk of fatty liver [21]:

$$ {\text{FLI}}\, = \,({\text{e}}^{{0.{953}*{\text{loge }}\left( {{\text{triglycerides}}} \right) + 0.{139}*{\text{BMI}} + 0.{718}*{\text{loge }}\left( {{\text{ggt}}} \right) + 0.0{53}*{\text{waist circumference}} - {15}.{745}}} ) \, / \, ({1}\, + \,{\text{e}}^{{0.{953}*{\text{loge }}\left( {{\text{triglycerides}}} \right) + 0.{139}*{\text{BMI}} + 0.{718}*{\text{loge }}\left( {{\text{ggt}}} \right) + 0.0{53}*{\text{waist circumference}} - {15}.{745})}} *{ 1}00. $$

Participants with FLI ≥ 60 were classified at high risk and participants with values < 30 were at low risk for fatty liver. Values between 30 and 60 were undetermined risk. Viral hepatitis B with HBs antigen and hepatitis C with anti-HCV, were measured by electrochemiluminescence technique.

## Evaluation of liver steatosis and fibrosis

Participants underwent two hepatic image methods, ultrasound (US) and liver transient elastography (TE), within an interval of up to six months. US was performed by a radiologist after 6 h of fasting. Steatosis was detected through observation of diffuse hyperechogenicity of the liver in comparison to kidneys, attenuation of ultrasound beam, and difficulty in visualizing intrahepatic vessels [22]. TE was performed with FibroScan® 502 (Echosens, Paris, France) by an experienced hepatologist, after participants fasted for 2 to 4 h. XL probe was selected for participants with BMI > 30 kg/m^2^ and distance skin-liver capsule ≥ 25 mm. M probe was selected for remaining participants. Steatosis stage was defined by categories of controlled attenuation parameter (CAP): S0: CAP < 248 dB/m; ≥ S1: 248–267 dB/m; ≥ S2: 268–279 dB/m; ≥ S3: ≥ 280 dB/m [23]. Fibrosis status was defined by categories of liver stiffness measurement (LSM): F0-F1: LSM < 7.0 kPa; F2: 7.0–8.7 kPa; F3: 8.8–10.3 kPa; F4 > 10.3 kPa [24]. CAP results ≥ S1 were considered steatosis and TE results ≥ F2 were considered with significant fibrosis. All participants had at least 10 valid measurements, a success rate above 60% and interquartile range/median ratio for LSM under 30%. Both imaging investigators had no access to clinical and laboratory data from participants.

## Evaluation of metabolic syndrome

Metabolic syndrome (MS) was defined according to the International Diabetes Federation criteria [25]. Considering that all participants have diabetes, central obesity plus an additional factor was necessary for diagnosing MS: central obesity: WC ≥ 90 cm in South American men or ≥ 80 cm in South American women; triglycerides ≥ 150 mg/dl (1.7 mmol/l) or on drug therapy for elevated triglycerides; HDL < 40 mg/dl (1.03 mmol/l) in men or < 50 mg/dl (1.29 mmol/l) in women or on drug therapy for low HDL; elevated BP ≥ 130 × 85 mmHg or receiving antihypertensives.

## Statistical analysis

Continuous variables are expressed as means ± standard deviations or median [interquartile range]. Categorical variables are expressed as frequencies and percentages. Student’ t-tests or Mann–Whitney U test, Chi-square or Fisher’s exact test, were used when indicated.

First, we performed an exploratory analysis to describe the baseline characteristics of the study population. Second, we compared demographical, clinical and laboratory parameters of the following groups: altered US vs. normal US; altered TE vs. normal TE; and finally altered hepatic image (US and/or TE) vs. normal hepatic images. Spearman’s correlation was performed to evaluate which factors were correlated with CAP and LSM measurements. Adjustment was performed with multivariable logistic regression to determine which factors could be associated with the presence of steatosis (steatosis on US and/or steatosis ≥ S1 on TE) and this was the dependent variable in all models. Independent variables were chosen based on statistical significance on exploratory analysis or biological plausibility. In the first model of logistic regression, age, gender, HbA1c and MS were the independent variables. Second model was done to determine which of the components of MS had stronger association with steatosis. Age, HbA1c, WC, HDL and triglycerides were the independent continuous variables, and gender and hypertension were the independent categorical variables. Finally, the third model was similar to second model, but also included components of FLI as independent variables. Model fit was assessed through Hosmer and Lemeshow and Omnibus test. Nagelkerke R^2^ was calculated and odds ratio (OR) with 95% confidence interval (CI) were expressed as indicated. Differences were considered significant at two-sided *p* < 0.05. All statistical analysis was performed with Statistical Package for Social Sciences (SPSS) 24.0.

## Results

Ultimately, we recruited 103 participants. Overall, 6.8% (n = 8) were excluded. One patient had missing blood samples and two were misdiagnosed with T1D. Five participants had a diagnosis of hepatitis (two cases of hepatitis C and three of hepatitis B) and were referred to a hepatologist. A total of 95 patients were included in the final analysis.

## Baseline characteristics and prevalence of steatosis

The mean age was 39 ± 13 years, with disease duration of 21 ± 9 years, and 55 (57.9%) participants were female. Forty-eight (50.2%) participants declared to be non-Caucasian (Black or Brown). MS was present in 42 participants (44.2%) and 45 participants (47.4%) were found overweight or obese. The median for HbA1c was 8.6% [IQR 2.1]. Steatosis was diagnosed by ultrasound in 12 participants (12.6%) and, by TE, in 16 participants (16.8%) and only 5 (5.3%) had steatosis on both exams. Eight participants (8.4%) showed significant fibrosis. Data shown in Table 1.

**Table 1:** Baseline characteristics

N	95
Age, years	39 ± 13
Female, n (%)	55 (57.9)
*Self-reported color-race, n (%)*	
Caucasian	47 (49.5)
Black	16 (16.8)
Brown	32 (33.7)
Years of formal education	12 ± 3
Diabetes duration, years	21 ± 9
Overweight, n (%)	31 (32.6)
Obesity, n (%)	14 (14.7)
Metabolic syndrome, n (%)	42 (44.2)
Steatosis on ultrasound, n (%)	12 (12.6)
*Steatosis on TE, n (%)*	16 (16.8)
≥ S1	13 (12.6)
≥ S2	0 (0)
≥ S3	3 (3.2)
*Steatosis on ultrasound* + *TE*	5 (5.3)
Fibrosis ≥ F2 on TE, n (%)	8 (8.4)
F2	3 (3.1)
F3	3 (3.1)
F4	2 (2.1)
*Categories of FLI, n (%)* High risk Undetermined risk Low risk	16 (16.8%) 16 (16.8%) 63 (66.3%)
HbA1c (%) (mmol/mol)	8.6 [2.1] 70 [24]

Data are represented as means ± standard deviation, median [interquartile range] or as numbers (percentages); TE: transient elastography; FLI: fatty liver index; HbA1c: glycated hemoglobin. ≥ S1, ≥ S2, ≥ S3 correspond to stages of steatosis and F2, F3 and F4 correspond to stages of fibrosis, determined by TE

### Demographic, clinical, and laboratory parameters according to hepatic images results

We stratified participants according to results on US (altered vs. normal US) and TE (altered vs. normal TE). The group with altered US presented higher rates of MS and higher FLI. Among variables involved in diagnosis of MS, the group with altered US presented higher triglyceride levels and lower HDL in comparison to group with normal US. There was no difference in anthropometric measurements such as BMI and WC, HbA1c or use of medications. The group with altered TE had higher BMI, WC, HC, waist-to-hip ratio (WHR), FLI, systolic and diastolic blood pressure, higher rates of MS and hypertension, and higher triglycerides levels, in comparison to normal TE group. No other laboratory data differences were found between the two groups of TE. Data shown in Tables 2 and 3.

**Table 2:** Participants characteristics according to hepatic ultrasound results

	Altered US	Normal US	p value
*Demographical and clinical characteristics*			
N (%)	12 (12.6)	83 (87.4)	
Age, years	40 ± 13	37 ± 13	0.459
Female gender, n (%)	9 (75.0)	46 (55.4)	0.199
Non-Caucasian, n (%)	8 (66.7)	40 (48.2)	0.232
Years of school attendance	11 ± 3	12 ± 3	0.383
Diabetes duration, years	19 ± 9	21 ± 10	0.406
BMI, kg/m^2^	26.7 ± 3.4	25.2 ± 4.1	0.209
WC, cm	89.7 ± 10.0	85.6 ± 11.8	0.253
HC, cm	100.0 ± 7.3	99.3 ± 7.7	0.759
WHR	0.90 ± 0.08	0.86 ± 0.08	0.135
SBP, mmHg	128 ± 18	127 ± 16	0.767
DBP, mmHg	76 ± 9	77 ± 11	0.668
Insulin dose, U/kg	0.82 ± 0.30	0.76 ± 0.31	0.499
Hypertension, n (%)	5 (41.7)	38 (45.8)	0.789
Anti-hypertensive use, n (%)	4 (33.3)	36 (43.4)	0.510
Metformin use, n (%)	2 (16.7)	9 (11.0)	0.567
Statin use, n (%)	5 (41.7)	40 (48.2)	0.672
Acetylsalicylic acid use, n (%)	2 (16.7)	16 (19.3)	0.829
Currently smoking, n (%)	2 (16.7)	4 (4.8)	0.165
Metabolic syndrome, n (%)	10 (83.3)	33 (39.8)	**0.005**
FLI	38 [43]	13 [35]	**0.028**
*Laboratory measurements*			
HbA1c (%) mmol/mol	8.6 [3.6] 70 [39]	8.6 [2.3] 70 [24]	0.757
FPG,mg/dl	116 [160]	160 [135]	0.728
Total cholesterol, mg/dl	154.5 [53.3]	167.0 [65.0]	0.787
HDL-c, mg/dl	38.1 [21.5]	50.0 [30.0]	**0.034**
LDL-c, mg/dl	83.6 [58.5]	94.8 [41.2]	0.375
Triglycerides, mg/dl	139.0 [190.8]	73.0 [60.8]	**0.028**
eGFR, ml/min/1.73 m^2^	107 [43]	99 [30]	0.728
Albumin, mg/dl	3.7 ± 0.6	4.0 ± 0.6	0.075
ALT, U/l	11.5 [15.3]	9.0 [7.0]	0.719
AST, U/l	15.5 [14.3]	13.0 [8.0]	0.507
GGT, mg/dl	18.5 [14.3]	19.0 [16.0]	0.848
CPK, mg/dl	100.5 [125.3]	81.0 [86.0]	0.670
CRP, mg/dl	0.4 [0.6]	0.2 [0.4]	0.334
Uric acid, mg/dl	3.6 [0.9]	3.6 [2.0]	0.848

Altered US refers to steatosis on hepatic ultrasound. Data are represented as means ± standard deviation, median [interquartile range] or as numbers (percentages). BMI: body mass index; WC: waist circumference, HC: hip circumference; WHR: waist-to-hip ratio; SBP: systolic blood pressure, DBP: diastolic blood pressure; HbA1c: glycated hemoglobin; FPG: fasting plasma glucose; HDL: HDL cholesterol; LDL: LDL cholesterol; eGFR: estimated glomerular filtration rate by CKD-EPI equation; ALT: alanine aminotransferase; AST: aspartate aminotransferase; GGT: gamma-glutamyl transferase; CPK: creatine phosphokinase; CRP: C reactive protein; FLI: fatty liver index

**Table 3:** Participants characteristics according to transient elastography results

	Altered TE	Normal TE	p value
*Demographical and clinical characteristics*			
N (%)	22 (23.2)	73 (76.8)	
Age, years	40 ± 11	39 ± 14	0.625
Female gender, n (%)	11 (50.0)	44 (60.3)	0.392
Non-Caucasian, n (%)	9 (40.9)	39 (53.4)	0.303
Years of school attendance	12 ± 4	12 ± 3	0.833
Diabetes duration, years	22 ± 10	20 ± 9	0.448
BMI, kg/m^2^	28.9 ± 3.7	24.3 ± 3.6	** < 0.001**
WC, cm	94.9 ± 11.3	83.2 ± 10.4	** < 0.001**
HC, cm	104.5 ± 7.4	97.8 ± 7.0	** < 0.001**
WHR	0.91 ± 0.08	0.85 ± 0.07	**0.003**
SBP, mmHg	135 ± 16	125 ± 16	**0.011**
DBP, mmHg	81 ± 11	76 ± 10	**0.027**
Insulin dose, U/kg	0.75 ± 0.26	0.77 ± 0.33	0.768
Hypertension, n (%)	14 (63.6)	29 (39.7)	**0.048**
Anti-hypertensive use, n (%)	13 (59.1)	26 (35.6)	0.050
Metformin use, n (%)	5 (22.7)	6 (8.2)	0.120
Statin use, n (%)	14 (63.6)	30 (41.1)	0.063
Acetylsalicylic acid use, n (%)	6 (27.3)	12 (16.4)	0.351
Currently smoking, n (%)	1 (4.5)	5 (5.3)	1.000
Metabolic syndrome, n (%)	15 (68.2)	27 (37.0)	**0.010**
FLI	60 [58]	13 [21]	** < 0.001**
*Laboratory measurements*			
HbA1c (%) mmol/mol	8.9 [2.8] 74 [29]	8.5 [2.3] 70 [24]	0.717
FPG,mg/dl	130 [200]	116 [131]	0.517
Total cholesterol, mg/dl	170.0 [77.3]	161.0 [65.0]	0.880
HDL-c, mg/dl	50.8 [24.5]	47.6 [32.8]	0.880
LDL-c, mg/dl	99.9 [46.1]	91.4 [40.2]	0.383
Triglycerides, mg/dl	89.0 [99.0]	75.0 [62.5]	**0.040**
eGFR, ml/min/1.73 m^2^	99 [30]	100 [29]	0.771
Albumin, mg/dl	4.0 ± 0.7	4.0 ± 0.6	0.992
ALT, U/l	10.0 [8.5]	8.0 [7.0]	0.260
AST, U/l	14.5 [8.3]	12.0 [7.5]	0.082
GGT, mg/dl	20.5 [41.3]	19.0 [16.5]	0.596
CPK, mg/dl	119.0 [172.0]	80.0 [86.0]	0.667
CRP, mg/dl	0.3 [0.9]	0.2 [0.4]	0.771
Uric acid, mg/dl	3.8 [1.8]	3.5 [1.7]	0.383

Altered TE refers to steatosis and/or fibrosis on transient elastography (TE). Data are represented as means ± standard deviation, median [interquartile range] or as numbers (percentages). BMI: body mass index; WC: waist circumference, HC: hip circumference; WHR: waist-to-hip ratio; SBP: systolic blood pressure, DBP: diastolic blood pressure; HbA1c: glycated hemoglobin; FPG: fasting plasma glucose; HDL: HDL cholesterol; LDL: LDL cholesterol; eGFR: estimated glomerular filtration rate by CKD-EPI equation; ALT: alanine aminotransferase; AST: aspartate aminotransferase; GGT: gamma-glutamyl transferase; CPK: creatine phosphokinase; CRP: C reactive protein; FLI: fatty liver index

When we considered both exams together, the group with altered hepatic image (US and/or TE) had higher BMI, WC, HC, WHR, FLI, and triglycerides, in comparison to the group with normal images. The rate of MS was higher in the group with altered images compared to the group with normal images. There was no difference in other measurements, including HbA1c, transaminases, insulin dose or other medications. Data shown in Table 4.

**Table 4:** Participants characteristics according to hepatic image results combined (US + TE)

	Altered hepatic image	Normal hepatic image	p value
N	30	65	
*Clinical variables*			
Age, years	39 ± 12	39 ± 13	0.995
Female, n (%)	18 (60.0)	37 (56.9)	0.778
Non-Caucasian, n (%)	15 (50.0)	33 (50.8)	0.994
Years of formal education	12 ± 4	12 ± 3	0.498
Diabetes duration, years	21 ± 10	20 ± 9	0.738
BMI, kg/m^2^	27.9 ± 3.9	24.2 ± 3.6	** < 0.001**
Waist circumference, cm	92.1 ± 11.2	83.1 ± 10.8	** < 0.001**
Hip circumference, cm	102.4 ± 7.9	97.9 ± 7.1	**0.006**
Waist-to-hip ratio	0.90 ± 0.08	0.85 ± 0.08	**0.003**
Systolic blood pressure, mmHg	131 ± 16	125 ± 16	0.092
Diastolic blood pressure, mmHg	79 ± 11	76 ± 10	0.297
Metabolic syndrome, n (%)	21 (67.7)	21 (30.0)	**0.001**
Insulin dose, U/kg	0.78 ± 0.28	0.76 ± 0.32	0.741
Anti-hypertensive use, n (%)	15 (50.0)	24 (36.9)	0.228
Metformin use, n (%)	5 (16.7)	6 (9.2)	0.292
Statin use, n (%)	16 (53.3)	28 (43.1)	0.351
Acetylsalicylic acid use, n (%)	7 (23.3)	11 (16.9)	0.459
Currently smoking, n (%)	3 (10.0)	3 (4.6)	0.316
*Laboratory measurements*			
HbA1c, % mmol/mol	8.9 [3.0] 74 [32]	8.5 [2.2] 69 [21]	0.717
Fasting plasma glucose, mg/dl	130 [175]	116 [137]	0.880
Total cholesterol, mg/dl	168.5 [62.8]	161.0 [66.0]	0.771
HDL cholesterol, mg/dl	45.8 [26.0]	48.6 [32.0]	0.880
LDL cholesterol, mg/dl	95.8 [51.7]	92.6 [39.6]	0.383
Triglycerides, mg/dl	103.0 [103.8]	72.0 [61.5]	**0.040**
eGFR, ml/min/1.73 m^2^	103 [32]	99 [28]	0.771
Albumin, mg/dl	3.9 ± 0.7	4.0 ± 0.6	0.268
ALT, U/l	10.0 [11.3]	8.0 [6.5]	0.260
AST, U/l	14.5 [11.5]	12.0 [6.0]	0.082
GGT, U/l	19.5 [17.3]	19.0 [17.5]	0.830
CPK, U/l	100.5 [142.3]	81.0 [85.5]	0.667
C-reactive protein, mg/dl	0.3 [0.7]	0.2 [0.4]	0.771
Uric acid, mg/dl	3.8 [1.5]	3.5 [2.0]	0.383
Fatty liver index	46 [52]	11 [21]	** < 0.001**
*TE measurements*			
CAP, dB/m	234 ± 51	174 ± 33	** < 0.001**
LSM, kPa	5.6 [3.9]	4.8 [1.8]	0.276

Altered image refers to steatosis on ultrasound and/or steatosis and/or fibrosis on transient elastography (TE). Data are represented as means ± standard deviation, median [interquartile range] or as numbers (percentages). BMI: body mass index; HbA1c: glycated hemoglobin; eGFR: estimated glomerular filtration rate; ALT: alanine aminotransferase; AST: aspartate aminotransferase; GGT: gamma-glutamyl transferase; CPK: creatine phosphokinase; CAP: controlled attenuation parameter; LSM: liver stiffness measurement

Also, a sub-analysis showed that participants with altered hepatic image without MS (n = 9) had higher levels of HbA1c (9.5% [IQR 1.5] vs. 8.5% [IQR 2.8]; *p* = 0.028) and higher BMI (25.4 ± 3.47 kg/m^2^ vs. 22.6 ± 2.6 kg/m^2^; *p* = 0.01) in comparison to participants without altered images and without MS (n = 41). However, in multivariable analysis, only BMI was associated (OR: 1.42, 95% CI 1.07–1.89; *p* = 0.016) with altered image in the group without MS.

In Spearman’s correlation we found that CAP was directly correlated with BMI (ρ 0.369; *p* < 0.001), WC (ρ 0.370; *p* < 0.001), HC (ρ 0.343; *p* = 0.001), WHR (ρ 0.248; *p* = 0.016) and FLI (ρ 0.361, *p* < 0.001). No laboratory parameters were correlated with CAP. Also, LSM was directly correlated with BMI (ρ 0.262; *p* = 0.010), WC (ρ 0.229; *p* = 0.026) and WHR (ρ 0.204; *p* = 0.047) and inversely correlated with HDL (ρ − 0.360; *p* < 0.001).

### Descriptive data of the group with altered hepatic image

We explored the characteristics of the 30 participants who had either altered ultrasound and/or altered TE. Refer to Table 5 for detailed information.

**Table 5:** Descriptive data on participants with altered hepatic image

ID	Gender	Age, years	Diabetes duration, years	HbA1c % (mmol/mol)	Transaminases	Steatosis on ultrasound	Transient elastography	Metabolic syndrome components	FLI Risk
*Both images altered (US and TE)*									
1	Male	32	16	9.6 (81)	Normal	**Mild**	F0-F1 ** > S1**	Yes; triglycerides, HDL	**High**
5	Female	41	22	11.3 (100)	Normal	**Mild**	F0-F1 ** > S1**	Yes; HDL	Low
42	Female	35	13	7.3 (56)	Normal	**Mild**	**F3** S0	Yes; hypertension, HDL	Undetermined
46	Female	37	20	9.2 (77)	Normal	**Mild**	F0-F1 ** > S3**	Yes; hypertension, triglycerides	**High**
48	Male	51	25	8.0 (64)	Normal	**Mild**	F0-F1 ** > S1**	Yes; hypertension, triglycerides, HDL	**High**
*Altered US*									
6	Male	41	15	6.7 (50)	Normal	**Mild**	F0-F1 S0	Yes; triglycerides	Undetermined
7	Female	45	18	7.4 (57)	**ALT = 32 U/l** **AST = 50 U/l**	**Mild**	F0-F1 S0	Yes.; hypertension, triglycerides, HDL	Undetermined
27	Female	51	36	6.8 (51)	Normal	**Mild**	F0-F1 S0	Yes; hypertension, HDL	Low
33	Female	19	6	14.3 (133)	Normal	**Mild**	F0-F1 S0	Yes; HDL	Low
63	Female	51	31	9.5 (80)	Normal	**Mild**	F0-F1 S0	No	Low
89	Female	17	8	7.5 (58)	Normal	**Mild**	F0-F1 **S0**	Yes; HDL	Undetermined
96	Female	20	13	14.1 (131)	Normal	**Mild**	F0-F1 S0	No	Low
*Altered TE*									
15	Male	51	23	7.8 (62)	Normal	No	**F4** ** > S1**	Yes; hypertension	**High**
8	Male	21	18	10.3 (89)	Normal	No	F0-F1 ** > S1**	No	Low
18	Male	50	20	11.7 (104)	Normal	No	F0-F1 ** > S1**	Yes; hypertension	**High**
25	Female	30	16	7.2 (55)	Normal	No	F0-F1 ** > S1**	Yes; HDL	Low
35	Male	46	35	7.1 (54)	Normal	No	F0-F1 ** > S3**	Yes; hypertension	Undetermined
36	Female	26	17	9.6 (81)	Normal	No	F0-F1 ** > S1**	No	Low
44	Male	61	57	7.3 (56)	Normal	No	F0-F1 ** > S1**	Yes; hypertension, triglycerides, HDL	**High**
50	Male	50	10	8.9 (74)	Normal	No	F0-F1 ** > S1**	No	Low
53	Female	34	18	8.9 (74)	Normal	No	F0-F1 ** > S1**	No	**High**
58	Female	24	12	9.3 (78)	Normal	No	F0-F1 ** > S1**	No	**High**
84	Female	56	27	7.2 (55)	Normal	No	F0-F1 ** > S1**	Yes; hypertension	**High**
91	Female	28	23	10.4 (90)	Normal	No	F0-F1 ** > S3**	Yes; hypertension, HDL	Low
31	Male	33	27	8.4 (68)	Normal	No	**F4** S0	No	Low
34	Female	45	32	9.5 (80)	Normal	No	**F2** S0	Yes; hypertension	Undetermined
45	Male	48	11	10.4 (90)	Normal	No	**F2** S0	No	Low
70	Male	48	21	8.2 (66)	Normal	No	**F2** S0	Yes; hypertension	Low
73	Female	35	23	8.6 (70)	Normal	No	**F3**	Yes; hypertension, triglycerides, HDL	**High**
87	Female	43	18	11.2 (99)	Normal	No	**F3** S0	Yes; hypertension, Trig, HDL	**High**

ID: identification number on database; HbA1c: glycated hemoglobin A1c; FLI risk: fatty liver index risk. High risk corresponds to FLI ≥ 60; low risk < 30; undermined risk: FLI values between 30 and 60. S0, ≥ S1, ≥ S2, ≥ S3 correspond to stages of steatosis and F0-F1, F2, F3 and F4 correspond to stages of fibrosis, determined by elastography

Of the 30 participants, 21 (70.0%) had MS. Other than diabetes and WC, the most frequent component of MS was hypertension (n = 14/21), followed by low HDL (n = 13/21) and high triglycerides (n = 8/21).

Twelve (40.0%) participants had steatosis on ultrasound and sixteen (53.3%) had steatosis on TE. Five (16.7%) participants had both exams altered.

One patient (3.3%) in the altered hepatic image group had elevated transaminases and this was associated with mild steatosis on ultrasound but with normal TE.

Considering FLI results, eleven (36.7%) participants had high risk, thirteen (43.3%) had low risk and six (20.0%) had undetermined risk in the group with altered image. Out of those eleven with high risk, eight (72.7%) had altered TE and three (27.3%) had both images altered. Out of those thirteen with low risk, four (30.8%) had altered US only, eight (61.5%) had altered TE and only and one (7.7%) had both US and TE altered. Out of those six with undetermined risk, one (16.7%) had both US and TE altered, three (50.0%) had altered US only, and two (33.3%) had with altered TE only.

Eight participants had significant fibrosis (≥ F2) on TE with normal liver function tests and were referred to further investigation in the hepatology unit. One (12.5%) of them had mild steatosis on US; the others had normal US and normal CAP on TE. Also, six (75%) of those participants with fibrosis had MS. We performed a sub-analysis comparing the group with fibrosis vs. no fibrosis. Participants with fibrosis had higher WC (95.3 ± 12.7 cm vs. 85.1 ± 11.2 cm; *p* = 0.017), HC (104.8 ± 9.7 cm vs. 98.8 ± 7.3; *p* = 0.034) and BMI (29.6 ± 4.9 kg/m^2^ vs. 24.0 ± 3.8 kg/m^2^; *p* = 0.002). There was no difference between groups of fibrosis regarding other clinical and laboratory measurements.

### Multivariable logistic regression evaluating associated factors for steatosis by either imaging method

The first model of logistic regression confirmed the association between MS and steatosis on either hepatic image. Nagelkerke R^2^ was 16.7% and X^2^ was 11.22. Gender, age and HbA1c were not associated to steatosis. In the second model, triglycerides levels were the component of MS associated with risk of steatosis. Second model had a Nagelkerke R^2^ of 28% and X^2^ was 19.70. In the third model, triglycerides remained as the only risk factor for steatosis, Nagelkerke R^2^ was 32.1% and X^2^ was 22.97. Results are shown in Table 6.

**Table 6:** Multivariable logistic regression for evaluating associated factors for steatosis on hepatic image by either method (ultrasound and/or transient elastography)

Variable	B	Odds ratio	95% confidence interval	p value
*Model 1*				
Age, years	− 0.02	0.98	0.93–1.04	0.270
Female	0.04	1.04	0.36–2.96	0.943
HbA1c (%)	− 0.02	0.98	0.89–1.07	0.671
Metabolic syndrome	1.71	5.53	1.84–16.6	**0.002**
*Model 2*				
Age (years)	− 0.03	0.97	0.92–1.03	0.338
Female	− 0.50	0.61	0.19–1.88	0.386
HbA1c (%)	− 0.03	0.97	0.82–1.16	0.773
WC (centimeters)	0.04	1.04	0.99–1.10	0.102
HDL (mg/dl)	− 0.01	0.82	0.97–1.02	0.818
Triglycerides (mg/dl)	0.01	1.01	1.00–1.02	**0.015**
Hypertension	0.55	1.72	0.44–6.75	0.434
*Model 3*				
Age (years)	− 0.02	0.98	0.93–1.04	0.614
Female	− 0.43	0.65	0.20–2.16	0.485
HbA1c (%)	− 0.02	0.98	0.82–1.17	0.850
WC (centimeters)	− 0.02	1.01	0.92–1.10	0.883
HDL (mg/dl)	− 0.01	0.99	0.96–1.02	0.559
Triglycerides (mg/dl)	0.01	1.01	1.00–1.02	**0.012**
Hypertension	0.22	1.25	0.28–5.45	0.770
BMI (kg/m^2^)	0.19	1.21	0.94–1.57	0.134
GGT (mg/dl)	0.01	1.01	0.99–1.02	0.785

Steatosis on hepatic image was the dependent variable in all models. Independent variables were chosen based on statistical significance on exploratory analysis or biological plausibility. Model 1—Adjusted for age, gender, glycated hemoglobin (HbA1c) and metabolic syndrome. Model 2—Adjusted for age, gender, HbA1c, waist circumference (WC), HDL-cholesterol (HDL), triglycerides and hypertension. Model 3—Adjusted for age, gender, HbA1c, components of metabolic syndrome (WC, HDL-c, triglycerides and hypertension) and components of fatty liver index [WC, triglycerides, body mass index (BMI) and gamma-glutamyl transferase (GGT)]

## Discussion

In our study, prevalence of steatosis was 12.6% when ultrasound was used and 16.8% when TE was used. When we combined both imaging methods, altered results were associated with higher rates of MS, FLI and anthropometric measurements such as BMI and WC. The components of MS associated to steatosis were triglycerides, after multiple adjustment logistic regression.

The pathogenesis of NAFLD in T1D is controversial. Physiologically, pancreatic insulin is partly cleared in first-pass metabolism on liver, resulting in higher portal insulin levels and lower levels in peripheral circulation [18]. Portal hyperinsulinemia is associated with insulin resistance and stimulates lipogenesis and steatosis [3]. In T1D, because insulin is administered exogenously, this gradient is altered, which could protect against NAFLD [18]. However, alternative pathways have been proposed to explain NAFLD in T1D. ChREBP (Carbohydrate sensitive response element-binding protein) and SREBP-1c (Sterol regulatory element-binding protein 1) are transcription factors that can be activated in the presence of hyperglycemia, independently of hepatic insulin levels, leading to expression of lipogenic genes and promoting fatty liver [3, 18]. Also, lipoprotein disturbances (such as glycation of apolipoproteins and increased LDL oxidation) may be present in T1D and could result in reduced hepatic exportation of VLDL, leading to NAFLD [18]. These metabolic abnormalities can be present even in individuals with T1D and good glycemic control [26]. Few studies have investigated the prevalence of NAFLD in T1D, which ranges from 8 to 50%, depending on characteristics of the studied population such as age, frequency of obesity, ethnicity, and method for diagnosis of steatosis [11, 12, 19, 27–29]. To our knowledge, this is the first study to access prevalence of NAFLD in a sample of T1D in Brazil,, with different lifestyle, eating habits and different ethnicity.

Although FLI was initially developed in comparison to abdominal ultrasound, it has been compared to CAP on TE. One study reported that CAP performed better than FLI in detecting steatosis ≥ S2 on liver biopsy [30]. This study proposes a CAP cut-off of 310 dB/m to detect steatosis ≥ S2 but it analyzed a population different from ours: only 59% of participants had diabetes and mean BMI was 30 kg/m^2^. Therefore, this cut-off may not be applicable to our population. TE is widely used for prognosis assessment with fibrosis stage, but it is still there is still much discussion regarding optimal cut-off points for steatosis diagnosis through CAP [23], [1, 5]. Although ultrasound is the preferred initial image for detecting steatosis and TE is usually recommended for fibrosis assessment after steatosis was detected, we chose to perform both US and TE with CAP to see how the two methods would relate to each other [22, 31]. Although frequency of steatosis found with TE approximates to the frequency found with US, the two imaging methods identified different participants. However, so far, cut-off values of CAP have not been proposed for T1D in comparison to liver biopsy, emphasizing the controversial aspects of this subject in T1D and the need for further studies.

A relevant proportion (31.6%) of our sample had alteration in at least one of the hepatic images and this warrants attention. Participants with altered hepatic images should be regularly examined, at least once a year, with a combination of methods (TE + US + FLI), in order to detect early progression of liver disease. Also, we should reinforce metabolic control and weight loss, a real challenge in routine clinical practice.

Our study has some limitations. As previously mentioned, we used two non-invasive methods to detect NAFLD. US is the main tool for screening NAFLD, easily accessible, with low cost, but operator-dependent and with limited sensitivity [31]. TE, the other method, is not usually applied as first-line exam for diagnosis of steatosis. Although we did not have histological confirmation of our findings, the gold-standard exam would be liver biopsy, which is invasive, susceptible to sampling error [11, 12, 27–29] and inappropriate for screening purposes of our study. Another limitation was the cross-sectional design of the study. Follow-up is necessary to determine how participants with altered hepatic image will evolve. Also, sample was not big. Patients were conveniently recruited in regular medical appointments but it was necessary higher frequency of attendance in order to participate in the study. Not all of them were willing to participate because of financial difficulties involving absence from work and transportation. Participation was voluntary, with no financial support for individual costs of each patient. Also, we had technical problems with unavailability of TE and some participants could not complete both hepatic exams.

As strengths of our study we have a sample of participants with T1D, representative of the Brazilian population, which were screened by two methods. The majority of studies with NAFLD in T1D performed only ultrasound [10, 13, 15, 19]. Some performed MRI and found lower rates of NAFLD, but this resource is not widely available, it is expensive and time-consuming and therefore less applicable for screening purposes [32, 33]. Also, to our knowledge, no former studies have been conducted determining CAP as well as fibrosis assessment in T1D so we present this data for the Brazilian population. We found two studies that used TE for fibrosis assessment in children and adolescents with T1D, but CAP is not mentioned [32, 33].

As previously mentioned, NAFLD may be a complication that deserves attention in the T1D population, as overweight and obesity are increasing and insulin resistance is more frequently found. However, best screening strategy is yet to be established in this population. As reported in the meta-analysis by Vries et al., there is no consensus on how to report NAFLD prevalence, which resulted in high heterogeneity of results [16]. In this study differences could not be attributed to HbA1c, diabetes duration or BMI, similar to ours. However, metabolic syndrome, our main risk factor, is not mentioned in this meta-analysis because it was not reported by all studies.

In conclusion, screening of NAFLD should be considered for T1D with MS and increasing levels of triglycerides, even within the normal range. Diagnosis of NAFLD should be accompanied of measurements to improve metabolic parameters. Further prospective studies are necessary to determine the best screening strategy and outcomes in T1D and also to investigate which factors are associated with fibrosis development.

## Data Availability

Data is available upon request to corresponding author.
